# Quantitative Profiling of Polar Metabolites in Herbal Medicine Injections for Multivariate Statistical Evaluation Based on Independence Principal Component Analysis

**DOI:** 10.1371/journal.pone.0105412

**Published:** 2014-08-26

**Authors:** Miaomiao Jiang, Yujiao Jiao, Yuefei Wang, Lei Xu, Meng Wang, Buchang Zhao, Lifu Jia, Hao Pan, Yan Zhu, Xiumei Gao

**Affiliations:** 1 Tianjin State Key Laboratory of Modern Chinese Medicine, Tianjin University of Traditional Chinese Medicine, Tianjin, China; 2 Research and Development Center of TCM, Tianjin International Joint Academy of Biotechnology and Medicine, Tianjin, China; 3 China Buchang Hospital for Cardio-Cerebral Vascular Disease, Xi'an, China; Macau University of Science and Technology, Macao

## Abstract

Botanical primary metabolites extensively exist in herbal medicine injections (HMIs), but often were ignored to control. With the limitation of bias towards hydrophilic substances, the primary metabolites with strong polarity, such as saccharides, amino acids and organic acids, are usually difficult to detect by the routinely applied reversed-phase chromatographic fingerprint technology. In this study, a proton nuclear magnetic resonance (^1^H NMR) profiling method was developed for efficient identification and quantification of small polar molecules, mostly primary metabolites in HMIs. A commonly used medicine, Danhong injection (DHI), was employed as a model. With the developed method, 23 primary metabolites together with 7 polyphenolic acids were simultaneously identified, of which 13 metabolites with fully separated proton signals were quantified and employed for further multivariate quality control assay. The quantitative ^1^H NMR method was validated with good linearity, precision, repeatability, stability and accuracy. Based on independence principal component analysis (IPCA), the contents of 13 metabolites were characterized and dimensionally reduced into the first two independence principal components (IPCs). IPC1 and IPC2 were then used to calculate the upper control limits (with 99% confidence ellipsoids) of χ^2^ and Hotelling T^2^ control charts. Through the constructed upper control limits, the proposed method was successfully applied to 36 batches of DHI to examine the out-of control sample with the perturbed levels of succinate, malonate, glucose, fructose, salvianic acid and protocatechuic aldehyde. The integrated strategy has provided a reliable approach to identify and quantify multiple polar metabolites of DHI in one fingerprinting spectrum, and it has also assisted in the establishment of IPCA models for the multivariate statistical evaluation of HMIs.

## Introduction

Metabolic profiling is essential to ensure the quality, consistency, safety and efficacy of herbal medicine products; especially for the injection dosage form. Water extraction or decoction is the most favored method of preparation of herbal medicine injections (HMIs). Saccharides, amino acids, organic acids, and other primary metabolites are unavoidably extracted along with targeted secondary metabolites during the process of HMIs, such as Qingkailing injection [1], Danshen injection [2], Guanxinning injection [3], and Shuxuetong injection [4]. In general, amino acids are considered to provide tonic activities and act as key regulators of nutrient metabolism, and polysaccharides are believed to be one of the most important constitutes in some herbal materials for pharmacological activities. Some monosaccharides have the suppressive effect on cell-mediated immune reactions [5]. However, these primary metabolites in HMIs are often ignored to detect and set corresponding quality criteria in China Pharmacopeia and national standard. Due to the strong polarity and hydrophilicity, conventional reversed-phase performance liquid chromatography (HPLC) fingerprint [6], [7]and other analytical methodologies, including anion exchange chromatography [8], gas chromatography [9], [10], and capillary electrophoresis [11], [12], are limited to separate and detect primary metabolites unless with complicated pretreatment, derivatization reagents and laborious preparation procedures. To delineate various class metabolites in HMIs, different types of fingerprints are necessary for a holistic quality evaluation, which are difficult to realize during the practical industry processing. Therefore, a simple and fast approach is required to be capable of detecting saccharides, amino acids, and organic acids, together with mainly bioactive secondary metabolites in one fingerprinting spectrum simultaneously.

Proton nuclear magnetic resonance (^1^HNMR) spectroscopy provides access to detect all proton-bearing compounds, almost irrespective of the chemical compound class. Because of the signal intensity directly proportional to the number of nucleus contributing to a specific resonance [13], ^1^H NMR method achieved the identification and quantification of metabolites in a one-step acquisition. With simple sample preparation, ^1^H NMR facilitates high-throughput analysis for metabolic studies and quality control of various food [14]–[17] and herbal materials [18]–[20]. As a universal technique with the simplicity and rapidity of implementation, ^1^H NMR has expansive prospect of application in profiling of polar metabolites of HMIs.

Since the complexity of chemical composition, HMIs cannot be completely represented by a limited number of certain bioactive compounds [21]. To extract the feature variables, principal component analysis (PCA) [22] and independent component analysis (ICA) [23] are classical tools to reduce the dimension of multivariate. The components (PCs) in PCA method are mutually orthogonal, while ICA method contains the components to be statistically independent [24]. ICA has been found to be a successful alternative to PCA in eliminating the overlapping information between the components [25]. However, ICA faces some limitations due to some instability [26], the choice of number of components to extract and high dimensionality [27]. As a consequence, independent principal component analysis (IPCA) was proposed by Yao et al. in 2012 [28] to use PCA as a pre-processing step to reduce the dimension of the data, and then use ICA as a denoising process of PCA to separate relevant information. On simulation studies and real data sets, IPCA offered a better visualization of the data than ICA and with a smaller number of components than PCA. Owing to the benefit to generate denoised the loading vectors, we attempted to employ IPCA method to construct χ^2^ and Hotelling T^2^ control charts [29] for multivariate statistical analysis.

Danhong injection (DHI) is a patent injection made from the extracts of *Radix Salviae Miltiorrhizae* and *Flos Carthami* [30]. It has been widely used for the prevention and treatment of cardiovascular and cerebrovascular diseases in clinic [31]–[33]. In our previous work, ultra-performance liquid chromatography (UPLC) coupled with UV detection was adopted to identify 11polyphenolic acids in DHI [34]. However, the total weight of identified constituents accounted for only a low proportion (about 10%) of the solid content in DHI.

In this study, we describe a strategy to detect more hydrophilic primary metabolites in DHI based on quantitative ^1^H NMR spectroscopy. The absolute concentration of identified metabolites was calculated by using internal standard method. Linearity, precision, repeatability, stability and accuracy were carried out to validate the method. The contents of polar metabolites were further evaluated by the multivariate analysis tool IPCA to establish χ^2^ and Hotelling T^2^ control charts.

## Materials and Methods

### Materials and Chemicals

Thirty-six batches of DHI manufactured in 2011, 2012 and 2013 were provided by Heze Buchang Pharmaceutical Co. Ltd (Heze, China). The standards of valine, threonine, alanine, pyroglutamate, procatechuic aldehyde and asparagine were purchased from the National Institute for Food and Drug Control (Beijing, China). Salvianic acid and procatechuic acid were obtained from Zhongxin Innova Laboratories (Tianjin, RP China). Succinate and malonate were obtained from Dr. Ehrenstorfer GmbH (Augsburg, Germany). Fructose and glucose were purchased from Sigma (Aldrich, America). Rutinose was purchased from Hazard Communication (Tokyo, Japan).The purities of the compounds were all above 98%, using NMR analysis. Deuterium oxide (D_2_O, 99.9%) and sodium 3-trimethylsilyl [2,2,3,3-*d*_4_] propionate (TSP) were purchased from Cambridge Isotope Laboratories (Miami, FL, USA). D_2_O was used as internal lock; TSP was as internal standard for chemical shift calibration and quantification.

### Ethics

No specific permission was required for the described field studies. The field locations are neither privately owned nor protected, and neither endangered nor protected species were involved.

### Sample preparation

All the injection samples were subjected to freeze-drying. The dried powders (18 mg) were accurately weighed and dissolved with 600 µL of D_2_O containing 0.58 mM TSP. Exactly 500 µL of sample solution was transferred into a standard 5 mm NMR tube (Vineland, NJ, USA). No buffer was used due to the stable pH of injection.

### NMR measurements

^1^H NMR spectra were acquired at 298 K on a Bruker AV III 600 MHz NMR spectrometer (600.23 MHz for proton frequency) with a 5 mm broadband BBFO probehead. All pulse sequences were from Bruker pulse program library. A standard one dimensional composite pulse sequence (zgcppr) was employed to suppress the residual water signal. The 90° pulse width was adjusted to about 13 µm for each sample. Sixty-four scans were collected into 32k data points using a spectral width of 12335 Hz, a relaxation delay of 1.0 s and an acquisition time of 2.66 s. A 0.3 Hz line-broadening function was applied to all spectra for Fourier transformation (FT) followed by phasing and baseline correction.

For proton signals assignment purposes, a set of two dimensional (2D) spectra, including ^1^H-^1^H correlation spectroscopy (COSY), ^1^H-^1^H total correlation spectroscopy (TOCSY), ^1^H *J*-resolved (*J*-res), and ^1^H-^13^C heteronuclear single quantum coherence (HSQC), were acquired for selected samples and processed with similar parameters as described previously [35].

Spin-lattice relaxation time (*T*_1_) values of the quantified protons of individual constituent and TSP were measured using a classical inversion recovery pulse sequence with 10 relaxation delays (*τ*) ranging from 0.01 to 20 s.

### Quantification of the metabolites

Because the intensity of a given ^1^H NMR signal is directly proportional to its contributing number of protons, the amount of metabolites in DHI can be measured by the signal areas of given metabolites and an internal reference with known concentration. Thirteen metabolites in DHI were selected for quantification. The important parameters for data acquisition and processing of ^1^H NMR spectra must be set appropriately to obtain accurate and precise measurements [36]. Most of all, the relaxation delay *τ* should be long enough to ensure complete relaxation for all the signals of interest.^1^H NMR measurements are done in a longer acquisition time by choosing *τ*≥5× longest *T*_1_. For shortening the acquisition time, ^1^H NMR spectra can be acquired in an incompletely relaxed condition, and the absolute concentrations should be calculated taking the *T*_1_ values in consideration [37]. With the effective magnetization reading pulse of 90°, the quantification of chemical constituents in this study can be performed by using the following equation: 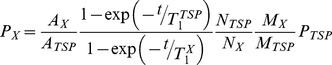
where *P_X_* and *P_TSP_* are the mass concentrations of metabolite and TSP, *A_X_* and *A_TSP_* are the integral areas for targeted signal of metabolite and for methyl groups of TSP, *N_X_* and *N_TSP_* are the proton numbers of metabolite and of methyl groups of TSP, *M_X_* and *M_TSP_* are the molar masses of metabolite and TSP, 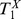 and 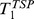 are the spin-lattice relaxation times for proton X and methyl protons of TSP, respectively; *t* is total relaxation time (relaxation delay plus acquisition time).

The quantitative ^1^H NMR method was checked for linearity, precision, repeatability, stability, and accuracy. Precision, repeatability and stability were calculated as the relative standard deviation (RSD). Recovery test was employed to determine the accuracy, and four typical compounds, alanine, glucose, salvianic acid and procatechuic aldehyde, were chosen to evaluate the average recovery.

### Assay for multivariate quality control

36 batches of DHI samples were split into two phases, Phase I and Phase II. Phase I as training set included 25 batches of qualified products manufactured continuously in the years of 2012 and 2013. Phase II as testing set included 6 batches of qualified products in 2012 and 2013, and 5 batches of expired products manufactured in 2011.

The quantitative resulting data of 13 metabolites was imported to R 3.0.2 software loaded with packages of MVA, MSQC and mixOmics (www.r-project.org) for multivariate statistical analysis. For simplifying the multivariate problem, principal component analysis (PCA) and independence principal component analysis (IPCA) were performed to reduce the dimensionality of data. The scores of principal components characterized the whole data were then imported to χ^2^ and hotelling T^2^ control charts to calculate the upper control limits (with 99% confidence ellipsoids). Moreover, as one of the requisites in control chart is the independence of the data, the independence of selected components in PCA and IPCA models were validated by autocorrelation function (ACF). The out-of-control samples in Phase II were examined by the upper control limits achieved from Phase I.

## Result and Discussion

### Proton signal assignments and chemical identification

**Figure 1:**
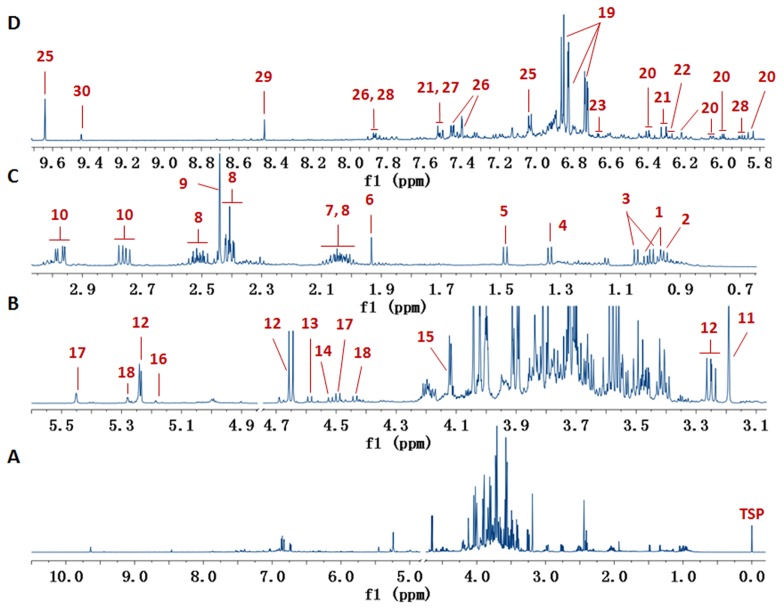
Representative ^1^H NMR spectra of Danhong injection. Peaks: 1, Isoleucine; 2, Leucine 3, Valine; 4, Threonine; 5, Alanine; 6, Acetate; 7, Proline; 8, Pyroglutamate; 9, Succinate; 10, Asparagine; 11, Malonate; 12, Glucose; 13, Galactose; 14, Arabinose; 15, Fructose 16, Rhamnose 17, Rutinose 18, Rutinulose; 19, Salvianic acid; 20, Salvianolic acid B; 21, Rosmarinic acid; 22, Lithospermic acid 23, Salvianolic acid A; 24, Procatechuic acid; 25, Procatechuic aldehyde; 26, 4-Hydroxybenzoic acid; 27, 4-Hydroxycinnamic acid; 28, Uridine; 29, Formate; 30, 5-(Hydroxymethyl)-2-furaldehyde.

A representative ^1^H NMR spectrum of DHI was shown in Fig.1. The resonance signals were assigned to 30 metabolites based on the elucidation with extensive 2D NMR experiments (^1^H-^1^H COSY, ^1^H-^1^H TOCSY, ^1^H *J*-resolved, HSQC),the literature data in our former work [35], and in-house database. In the range of *δ* 3.2–5.8, the spectrum is dominated by 5monosaccharides and 2 disaccharides, including glucose, galactose, arabinose, fructose, rhamnose, rutinose, and rutinulose. In the high-field region (*δ* 0.5–3.2), 8 amino acids (isoleucine, leucine, valine, threonine, alanine, proline, pyroglutamate, asparagine) and 3 organic acids (acetate, succinate, malonate) were observed. In the low-field region (*δ* 5.8–10.0), 7 polyphenolic acids, including salvianic acid, salvianolic acid B, salvianolic acid A, rosmarinic acid, lithospermic acid, procatechuic acid and procatechuic aldehyde, together withuridine and 5-(hydroxymethyl)-2-furaldehyde (5-HMF), were identified. Moreover, 3 organic acids (4-hydroxybenzoic acid, 4-hydroxycinnamic acid, and formate) were observed as well in the low-field region. The chemical shifts of the identified 30 metabolites by^1^H NMR were listed in Table 1.To our knowledge, 7 saccharides and 6 organic acids were reported for the first time in DHI. Without the need of any sample pretreatment or pre-column derivatization, the established ^1^H NMR method provided an approach to determine 7 saccharides, 6 organic acids, 8 amino acids, 1nucleoside, 1 carbohydrate derivatives (5-HMF) and 7 polyphenolic acids in DHI simultaneously.

**Table 1:** Chemical shifts for 30 metabolites identified in the ^1^H NMR spectra of Danhong injection.

Peak	Metabolites	Assignment	*δ* ^1^H (multiplicity^a^)	Assigned with
1	Isoleucine	*δ*-CH_3_, *γ′*-CH_3_, *γ*-CH_2_, *β*-CH, *α*-CH	0.96 (t, 6.1 Hz), 1.01 (d, 6.8 Hz), 1.26 (m), 1.47 (m), 1.96 (m), 3.66 (m)	COSY, TOCSY, *J*-res, HSQC
2	Leucine	*δ*, *δ′*-CH_3_, *β*-CH_2_,*γ*-CH	0.95 (m), 1.67 (m), 3.72 (m)	COSY, TOCSY, *J*-res, HSQC
3	Valine	*γ′*-CH_3_, *γ*-CH_3_, *β*-CH, *α*-CH	1.00 (d, 7.0 Hz), 1.05 (d, 7.0 Hz), 2.26 (m), 3.60 (d, 4.2 Hz)	COSY, TOCSY, *J*-res, HSQC
4	Threonine	*γ*-CH_3_, *β*-CH, *α*-CH	1.36 (d, 6.5 Hz), 4.25 (m), 3.58 (d, 6.4 Hz)	COSY, TOCSY, *J*-res, HSQC
5	Alanine	*β*-CH_3_, *α*-CH	1.48 (d, 7.5 Hz), 3.78 (m)	COSY, TOCSY, *J*-res, HSQC
6	Acetate	CH_3_	1.93 (s)	*J*-res, HSQC
7	Proline	*γ*-CH_2_, *δ*-CH_2_, *β*-CH_2_, *α*-CH	2.00 (m), 3.34 (m), 3.42 (m), 2.35 (m),4.14 (m)	COSY, TOCSY, *J*-res, HSQC
8	Pyroglutamate	*γ*-CH_2_, *β*-CH_2_, *α*-CH	2.04 (m), 2.51 (m), 2.41 (m), 4.18 (m)	COSY, TOCSY, *J*-res, HSQC
9	Succinate	CH_2_	2.44 (s)	*J*-res, HSQC
10	Asparagine	*β*-CH_2_, *α*-CH	2.76 (dd, 14.0, 8.2 Hz), 2.97 (dd, 14.0, 4.3 Hz), 4.00 (m)	COSY, TOCSY, *J*-res, HSQC
11	Malonate	CH_2_	3.19 (s)	*J*-res, HSQC
12	Glucose	*α*C_1_H, *α*C_2_H, *α*C_3_H, *α*C_4_H, *α*C_5_H, *β*C_1_H, *β*C_2_H, *β*C_3_H	5.24 (d, 3.6 Hz), 3.55 (m), 3.72 (m), 3.42 (m), 3.85 (m), 4.65 (d, 7.8 Hz), 3.25 (dd, 7.8, 9.2 Hz), 3.48 (m)	COSY, TOCSY, *J*-res, HSQC
13	Galactose	*α*C_1_H, *α*C_2_H, *α*C_3_H, *β*C_1_H, *β*C_2_H, *β*C_3_H, *β*C_4_H	5.27 (d, 3.8 Hz), 3.83 (m), 4.02 (m), 4.59 (d, 7.8 Hz), 3.50 (m), 3.65 (m), 3.93 (m)	COSY, TOCSY, *J*-res, HSQC
14	Arabinose	*α*C_1_H, *α*C_2_H, *α*C_3_H, *β*C_1_H, *β*C_2_H, *β*C_3_H	5.25 (d, 3.6 Hz), 3.82 (m), 4.02 (m), 4.52 (d, 7.8 Hz), 3.51 (m), 3.68 (m)	COSY, TOCSY, *J*-res, HSQC
15	Fructose	C_1_H, C_3_H, C_4_H, C_5_H, C_6_H	3.58 (m), 3.69 (m), 3.82 (m), 4.11 (m), 3.90 (m), 4.11 (m), 3.82 (m), 4.00 (m), 3.69 (m), 3.82 (m), 4.02 (m)	COSY, TOCSY, *J*-res, HSQC
16	Rhamnose	*α*C_1_H, *α*C_2_H, *α*C_3_H, *α*C_4_H, *α*C_5_H, *β*C_2_H, *β*C_3_H, *β*C_4_H, *β*C_5_H	5.18 (d, 1.7 Hz), 3.91 (m), 3.79 (m), 3.43 (m), 3.85 (m), 3.92 (m), 3.59 (m), 3.37 (m), 3.43 (m)	COSY, TOCSY, *J*-res, HSQC
17	Rutinose	Rha- C_1_H, Glc-C_1_H	5.42 (d, 1.7 Hz), 4.50 (d, 7.4 Hz)	COSY, TOCSY, *J*-res, HSQC
18	Rutinulose	Rha- C_1_H, Fru-C_1_H	5.25 (d, 1.7 Hz), 4.45 (d, 7.4 Hz)	COSY, TOCSY, *J*-res, HSQC
19	Salvianic acid	H-2, H-5, H-6, H_2_-7, H-8	6.81 (d, 1.5 Hz), 6.86 (d, 8.0 Hz), 6.74 (dd, 8.0, 1.5 Hz), 2.85 (m), 3.04 (m), 5.42 (m)	COSY, TOCSY, *J*-res, HSQC
20	Salvianolic acid B	H-2, H-3, H-5, H-6, H-*β*, H-*α*, H-5″, H-6″, H_2_-7″, H-8″, H-2″′, H-5″′, H-6″′, H-7″′, H-8″′	6.01 (d, 5.6 Hz), 4.33 (d, 5.6 Hz), 7.06, 6.92, 6.99 (d, 16.0 Hz), 5.85 (d, 16.0 Hz), 6.93, 6.76, 2.90 (m), 3.10 (m), 5.00 (m), 6.24 (d, 2.6), 6.42 (d, 8.0), 6.12 (dd, 8.0, 2.6 Hz), 2.98 (m), 2.56 (m), 4.90 (m)	COSY, TOCSY, *J*-res, HSQC
21	Rosmarinic acid	H-2, H-5, H-6, H-*β*, H-*α*, H-2′, H-5′, H-6′, H_2_-7′	6.98 (d, 1.5 Hz), 6.72 (d,8.0 Hz), 6.89 (dd, 8.0, 1.5 Hz), 7.49 (d, 16.0 Hz), 6.32 (d, 16.0 Hz), 6.90 (d, 1.5 Hz), 6.64 (d, 8.0 Hz), 6.55 (dd, 8.0, 1.5 Hz), 2.91 (m), 3.13 (m)	COSY, TOCSY, *J*-res, HSQC
22	Lithospermic acid	H-5, H-6, H-*β*, H-*α*, H-2′, H-5′, H-6′, H_2_-7′, H-8′, H-2″, H-5″, H-6″	6.52 (d, 8.5 Hz), 6.57 (d, 8.5 Hz), 7.22 (d, 16.0 Hz), 6.32 (d, 16.0 Hz), 6.74 (d, 1.5 Hz), 6.65 (d, 8.0 Hz), 6.52 (dd, 8.0, 1.5 Hz), 2.81 (m), 2.87 (m), 4.85 (m), 6.66 (d, 1.5 Hz), 6.55 (d, 8.0 Hz), 6.46 (dd, 8.0, 1.5 Hz)	COSY, TOCSY, *J*-res, HSQC
23	Salvianolic acid A	H-5, H-6, H-*β*, H-*α*, H-2′, H-5′, H-6′, H_2_-7′, H-8′, H-2″, H-5″, H-6″, H-7″, H-8″	6.71 (d, 8.0 Hz), 7.05 (d, 8.0 Hz), 7.86 (d, 16.0 Hz), 6.20 (d, 16.0 Hz), 6.66 (d, 1.5 Hz), 6.59 (d, 8.0 Hz), 6.48 (dd, 8.0, 1.5 Hz), 2.90 (m), 3.01 (m), 5.11 (dd, 8.5, 3.5 Hz), 7.00 (d, 1.0 Hz), 6.69 (d, 8.0 Hz), 6.82 (dd, 8.0, 1.5 Hz), 6.60 (d, 16.0 Hz), 7.08 (d, 16.0 Hz)	COSY, TOCSY, *J*-res, HSQC
24	Procatechuic acid	H-2, H-5, H-6	7.41 (d, 2.0 Hz), 6.80 (d, 8.0 Hz), 7.45 (dd, 8.0, 2.0 Hz)	COSY, TOCSY, *J*-res, HSQC
25	Procatechuic aldehyde	H-2, H-5, H-6, CHO	7.10 (d, 2.0 Hz), 6.80 (d, 8.0 Hz), 7.08 (dd, 8.0, 2.0 Hz), 9.63 (s)	COSY, TOCSY, *J*-res, HSQC
26	4-Hydroxybenzoic acid	H-3, 5, H-2, 6	7.83 (d, 8.8 Hz), 6.91 (d, 8.8 Hz)	COSY, TOCSY, *J*-res, HSQC
27	4-Hydroxycinnamic acid	H-3, 5, H-2, 6, H-*β*, H-*α*	7.54 (d, 9.0 Hz), 6.89 (d, 9.0 Hz), 7.60 (d, 16.0 Hz), 6.37 (d, 16.0 Hz)	COSY, TOCSY, *J*-res, HSQC
28	Uridine	H-5, H-6, Xyl-C_1_H	5.88 (d, 8.1 Hz), 7.88 (d, 8.1 Hz), 5.91 (d, 4.2 Hz)	COSY, TOCSY, *J*-res, HSQC
29	Formate	CHO	8.46 (s)	*J*-res, HSQC
30	5-(Hydroxymethyl)-2-furaldehyde	H-3, H-4, CHO	7.23 (d, 3.5 Hz), 6.51 (d, 3.5 Hz), 9.45 (s)	COSY, TOCSY, *J*-res, HSQC

a Multiplicity: singlet (s), doublet (d), triplet (t), doublet of doublets (dd), quintet (q), multiplet (m).

### Quantitative ^1^H NMR analysis and method validation

Due to the narrow chemical shift range of ^1^H NMR and frequent signal overlap, it is a challenge to quantify all the constituents in a mixture. In our study, 13 metabolites with fully separated signals were selected for quantification. In order to improve the efficiency of ^1^H NMR method, the spectra were acquired in an incompletely relaxed state. As a consequence, the spin-lattice relaxation time (*T*_1_) value must be accurately measured and taken into account for quantitative analysis. The *T*_1_ values were determined by the inversion-recovery experiments (Table 2). Accordingly, the absolute concentrations of the 13 metabolites were calculated from three parallel samples of each batch (Table S1 in File S1).

**Table 2:** Proton spin-lattice relaxation time (*T*_1_) values for 13 selected metabolites and the method validation parameters for ^1^HNMR method.

Metabolites	*δ*(ppm)	*T*_1_ (s) (*n* = 3)	Regression equation (*n* = 3)	*r* ^2^	Intra-day precision (*n* = 6) RSD (%)	Inter-day precision (*n* = 3) RSD (%)	Repeatability (*n* = 6) RSD (%)	Stability (*n* = 6) RSD (%)	Average recovery^a^ (%) ± SD (*n* = 3)
Valine	1.03 (3H)	0.944±0.049	y = 7.9917x - 0.1254	0.9996	0.79	0.84	1.83	1.67	-
Threonine	1.31 (3H)	1.323±0.102	y = 7.9895x - 0.1288	0.9995	0.49	0.98	1.94	0.99	-
Alanine	1.47 (3H)	1.451±0.120	y = 11.536x - 0.0821	0.9997	0.61	0.35	1.46	0.58	106.6±1.2
Pyroglutamate	2.41 (2H)	2.249±0.044	y = 3.9348x - 0.0387	0.9980	0.20	0.29	1.48	0.37	-
Succinate	2.42 (4H)	1.844±0.268	y = 9.7223x - 0.2087	0.9993	0.89	0.93	1.85	2.21	-
Asparagine	2.76 (1H)	0.562±0.019	y = 1.8370x - 0.1871	0.9979	0.60	1.49	2.75	1.78	-
Malonate	3.19 (2H)	2.116±0.136	y = 0.7533x - 0.0317	0.9972	0.20	0.56	1.66	1.43	-
Fructose	4.10 (3H)	3.303±0.210	y = 1.2045x - 0.2608	0.9998	0.45	0.34	1.69	2.06	-
Glucose	5.24 (1H)	1.684±0.206	y = 0.5486x - 0.0912	0.9997	0.32	0.68	2.59	1.32	106.6±2.5
Rutinose	5.44 (1H)	1.447±0.164	y = 2.4419x - 0.1850	0.9992	0.59	0.76	2.49	1.78	-
Salvianic acid	6.74 (1H)	1.843±0.139	y = 1.2845x - 0.1858	0.9994	0.23	0.34	2.01	1.23	98.3±2.1
Procatechuic acid	7.41 (1H)	3.542±0.326	y = 0.5172x - 0.0083	0.9983	0.67	0.93	1.93	1.99	-
Procatechuic aldehyde	9.63 (1H)	2.932±0.436	y = 1.2468x - 0.0013	0.9992	0.78	0.85	1.72	1.82	91.3±4.0

a Recovery (%) = [(found−original)/spiked]×100.

#### Linearity

^1^H NMR as method itself is linear and no calibration is necessary for the determination of molar ratios of mixtures. Thus, the 13 metabolites in five different molar ratios confirmed the linearity of NMR spectroscopy. Good linearity was achievable, as indicated by the equations and satisfactory correlation coefficients (*r* ^2^).

#### Precision

The intraday and interday precision was determined by analyzing six replicates on the same day and on three consecutive days respectively. The intraday precision for the contents of 13 metabolites ranged from 0.20% to 0.89%, and the interday precision ranged from 0.29% to 1.49%. The RSD values were adequate and indicated the suitability of the method.

#### Repeatability

Six samples prepared from the same batch showed RSD values ranging from 1.46% to 2.75%, indicating a high repeatability.

#### Stability

One sample was analyzed to determine stability on three consecutive days. The RSD values of the analytes were in the range of 0.37% to 2.21%.

#### Accuracy

Considering the limited volume of NMR tube and the cost of using deuterium reagents to dilute sample continuously, four metabolites of different types were employed, including alanine, glucose, salvianic acid and procatechuic aldehyde. The recovery was calculated as the ratio of the response of the selected four compounds in the spiked DHI samples against that of the standards at the same levels. The average recoveries were found to be 106.6% (±1.2), 106.6% (±2.5), 98.3% (±2.1) and 91.3% (±4.0) for alanine, glucose, salvianic acid and procatechuic aldehyde, respectively, indicating acceptable recovery.

**Figure 2:**
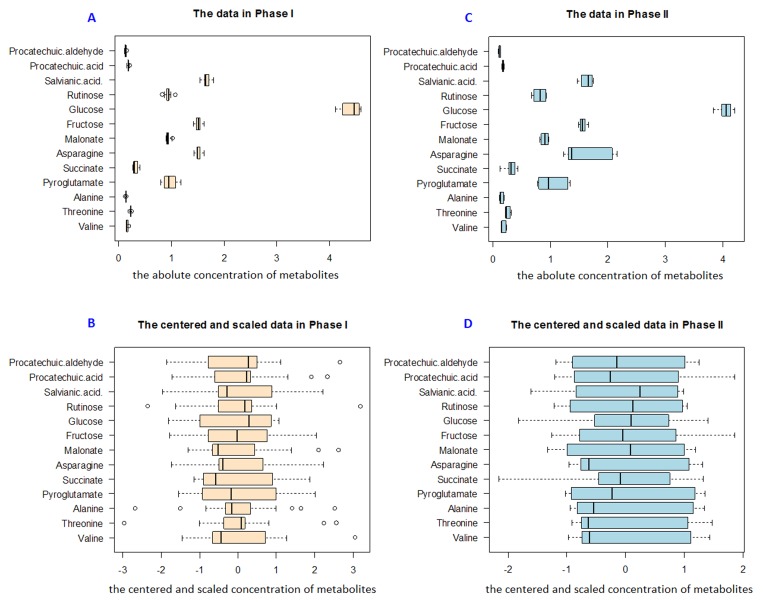
Quantitative data of selected 13 metabolites. Boxplots of the quantitative data before and after centering and scaling in Phase I (A, B) and Phase II (C, D).

According to the results, the concentration of glucose was extremely high in DHI as shown in Fig. 2A and 2B, and the amount of saccharides, amino acids and organic acids represented about 60% of the total solid content of DHI (Table S1 in File S1).

### Control Charts based on PCA and IPCA

**Figure 3:**
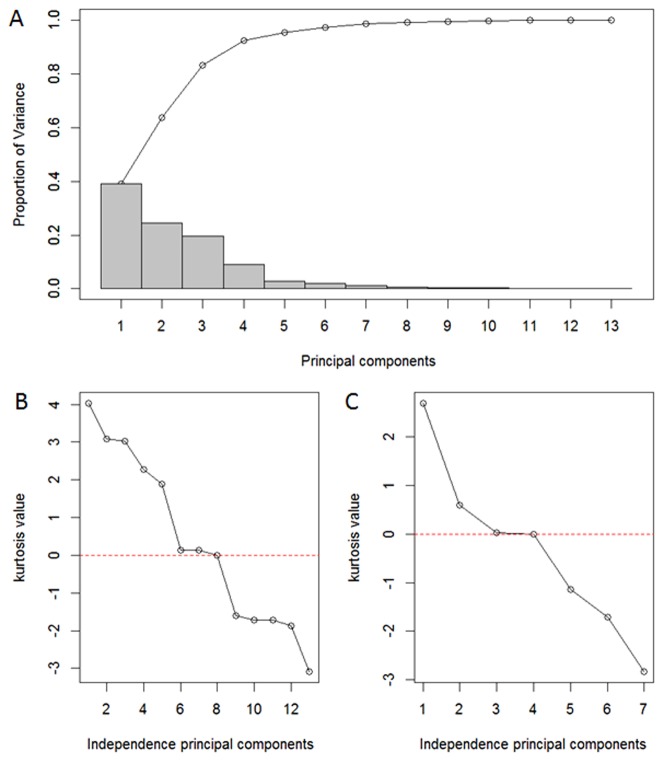
Component contributions of PCA and IPCA. (A) Pareto chart of principal components (PCs) in PCA indicated the first three PCs in Phase I explained 80.99% variances. (B) Kurtosis measurements of all extracted independence principal components (IPCs) in IPCA with Phase I data. The kurtosis of IPC8 was close to zero, so the first 7 components of IPCA were used to choose exact numbers of IPCs. (C) Kurtosis measurements of the first 7 IPCs showed that the kurtosis of IPC3 was close to zero, and the first 2 components were sufficient with IPCA.

**Figure 4:**
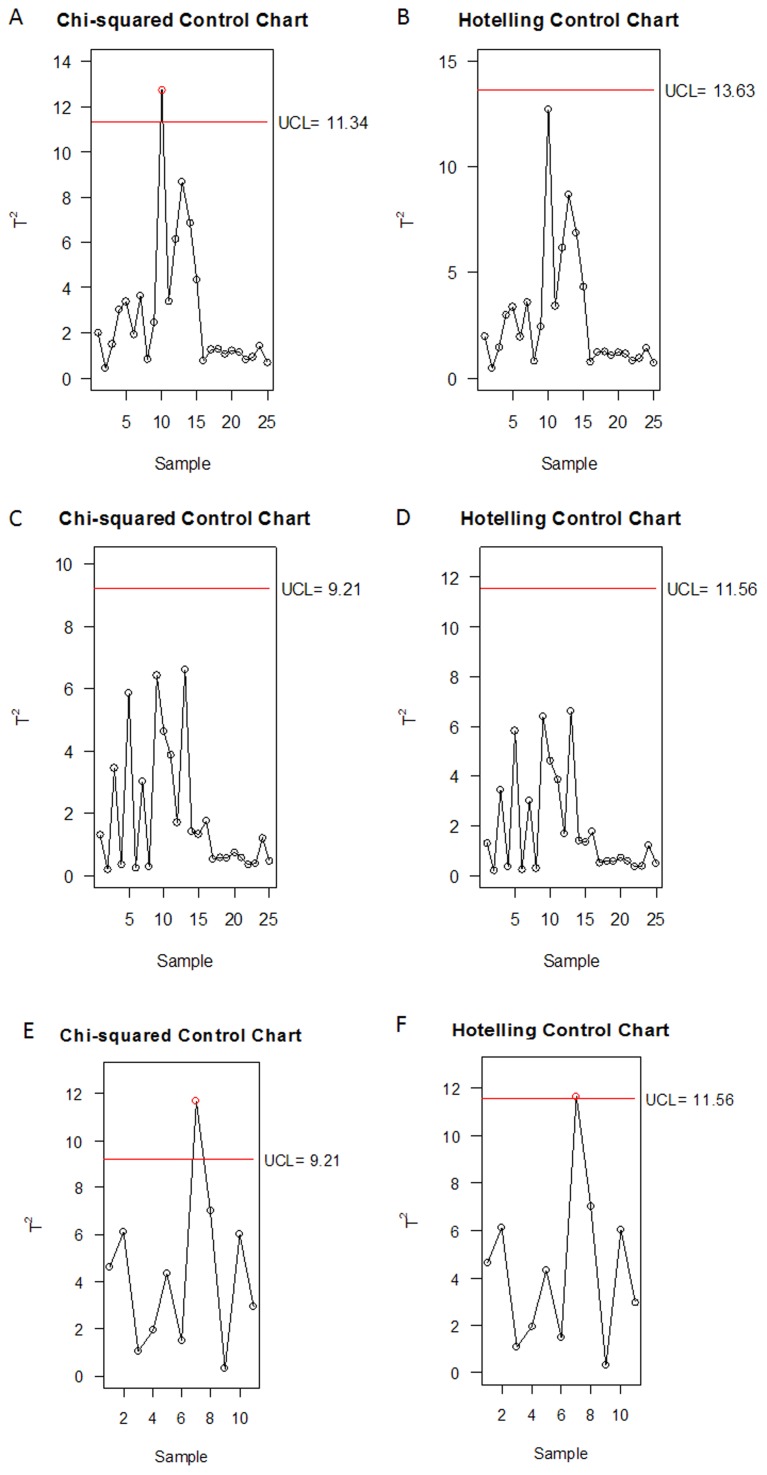
Multivariate quality control charts. The χ^2^ and Hotelling control charts of the first three principle components (PCs) in Phase I (A and B), and the first two independence principle components (IPCs) in Phase I (C and D) and Phase II (E and F). Red solid lines represented the upper control limits (UCL) of control charts with 99% confidence region.

**Figure 5:**
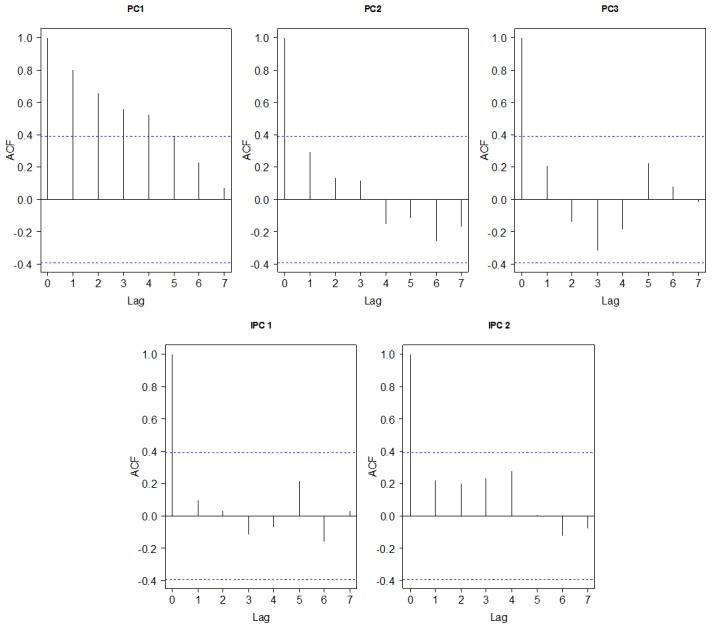
Autocorrelation function (ACF) validation of PCA and IPCA. Correlograms for the first three principle components (PC1, PC2, PC3) and the first two independence principle components (IPC1, IPC2) in Phase I. Blue dashed lines represented the confidence bands of ACF. PC1 fell outside of the confidence bands with autocorrelation or dependence, it was said that PCA model was not successful.

With the enhancement in quality control of DHI, the analysis should be performed through a multivariate approach, that is, the above 13 metabolites must be analyzed together, not independently. The concentrations of 13 metabolites were mean centered and unit-variance scaled before being analyzed by PCA and IPCA (Fig. 2C and 2D). To avoid the loss of significant information, the percent specified of the principal components (PCs) cumulative proportion of explain variance is normally fixed on 80% in PCA model [29]. Thus, the first three PCs in Phase I (Fig. 3A) were selected to construct χ^2^ (Fig. 4A) and Hotelling T^2^ (Fig. 4B) control charts. Since the independence of the data is one of the requisites in control chart, we assessed the marginal independence of each necessary PC to indicate the model validation [38]. Correlograms (Fig. 5 PC1-PC3) showed that PC1 fell outside of the confidence bands, which indicated that there was an evidence of autocorrelation or dependence of PC1, and PCA model using Phase I data was not achieved.

In order to remove the autocorrelation effects of PCA, we employed IPCA to generate denoised and independent loading vectors [28]. The kurtosis measure of loading vectors was used to decide the number of independent principal components (IPCs). The kurtosis of all extracted IPCs was plotted in Fig. 3B, whereas the kurtosis of IPC7 was close to zero. By using the first 7 components of IPCA, the exactly choosing number of IPCs was obtained (Fig. 3C). Since the kurtosis of IPC3 was close to zero, the first 2 components were sufficient with IPCA. The presence of autocorrelation was assessed as shown in Fig. 5 (IPC1 and IPC2), which indicated that there was no evidence of relation between the adjacent observations. Therefore the original 13 dimension of our data had been reduced to a two-dimensional problem.

**Figure 6:**
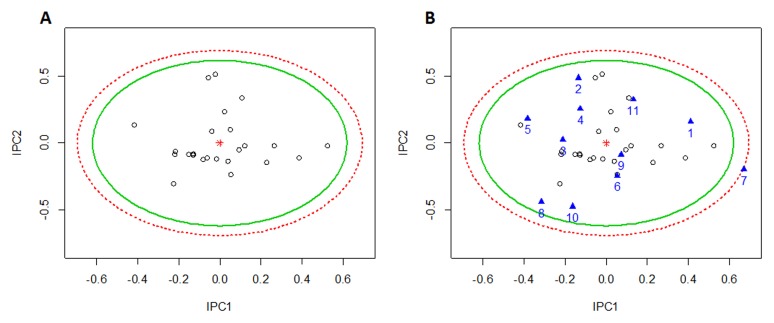
The confidence ellipses for quality control. Scatterplots for the first two IPC scores with the confidence ellipses in Phase I (A) and Phase II (B). The χ^2^ control ellipse used as process region was shown in green solid line, while the Hotelling T^2^ control ellipse used as tolerance region was in red dashed line. The numbers represented the samples in Phase II listing in Table S2 in File S1, and the seventh sample fell outside the tolerance region.

**Figure 7:**
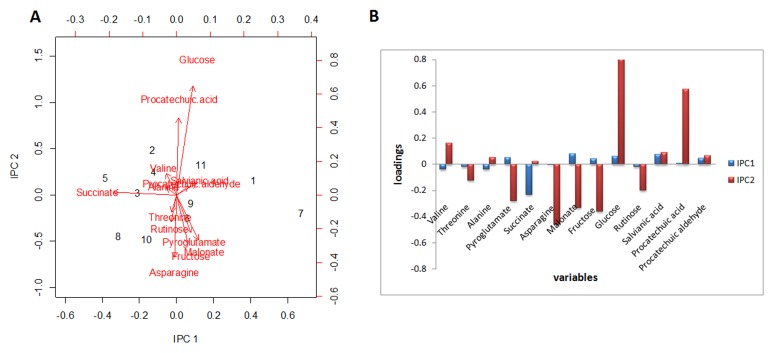
The loading plots and values of IPCA. (A) The IPCA loading plots for the first two IPCs in Phase II. The numbers represented the samples in Phase II listing in Table S2 in File S1. The influence of metabolites was showing in red arrow lines. (B) Bar diagram showed the loading values of metabolites in first two independence principle components.

Then the first two IPCs were taken to establish the in-control state (Phase I). According to χ^2^ (Fig. 4C) and Hotelling T^2^ (Fig. 4D) control charts with the 99% confidence region, the upper control limits (UCL) were determined as 9.21 and 11.56, respectively. The first two IPCs were consequently controlled through 2D ellipsoids (Fig. 6).The χ^2^ control ellipse (UCL = 9.21) could be used as process region, and the T^2^ control ellipse with less restrictive (UCL = 11.56) was used as tolerance region. In both cases, all the points in Phase I fell inside the confidence ellipsoids (Fig. 6A). The samples in Phase II (Table S2 in File S1) were monitored by employing the UCLs of both χ^2^ and T^2^ charts obtained from Phase I (Fig. 4E and 4F), and the points of Phase II were added into 2D ellipsoids (Fig. 6B). The seventh sample fell outside the 99th confidence ellipsoids of both the process and tolerance regions, indicating the presence of out-of-control sample (batch 110402). The decomposition of T^2^ value showed that the out-of-control variability was associated to the IPC1 since *p*-value was equal to 0.0032 (Table 3). The same result was also obtained through IPCA loading plots as shown in Fig. 7. Although the loading plot could not exactly determine which metabolites were responsible for the variation, it still showed the contents of succinate, malonate, glucose, fructose, salvianic acid and protocatechuic aldehyde made more contributions to the independent loading vectors of IPC1.

**Table 3:** Decomposition of the seventh sample in Phase II.

T^2^	Decomposition	UCL	*p*-Value
[1,]	10.7286	7.5731	0.0032
[2,]	0.9184	7.5731	0.3475
[3,]	11.6469	11.5641	0.0000

## Conclusion

Based on quantitative ^1^H NMR analysis, a reliable approach for simultaneous determination of amino acids, organic acids, saccharides, and botanic secondary metabolites of HMIs in one fingerprinting spectrum has been developed and validated by using Danhong injection as a model. The method had taken*T*_1_ values into account when calculated the contents of feature metabolites, which allowed the assay with good linearity, precision, repeatability, stability and accuracy. Unlike HPLC fingerprinting methods, the ^1^H NMR approach has the significant advantages of less analysis time (about 5 min) without chromatographic separation, and no requirement of standard materials used to quantitative analysis. In combination with IPCA, two kinds of multivariate control charts (χ^2^ and Hotelling T^2^) were also successfully carried out for detecting off-test HMI samples by employing the independence principle components. The decomposition of T^2^ value and IPCA loading vectors can reflect the significance variations of overall metabolite profiling, although it cannot decide the mutative metabolites exactly. The established multivariate models have the prospects for extracting sufficient characterization to monitor more feature metabolites in HMIs.

## Supporting Information

File S1 **Quantification of 13 chemical markers selected in the 1H NMR spectra of DHI samples in Phase I (Table S1) and Phase II (Table S2).**
(DOC)
