# Supplementary material for: Quantitative Profiling of Polar Metabolites in Herbal Medicine Injections for Multivariate Statistical Evaluation Based on Independence Principal Component Analysis
Source: PLoS One. 2014 Aug 26;9(8):e105412. doi: 10.1371/journal.pone.0105412 (PMC4144889; doi:10.1371/journal.pone.0105412)
Supplement: File S1 — Quantification of 13 chemical markers selected in the 1H NMR spectra of DHI samples in Phase I (Table S1) and Phase II (Table S2). (DOC) [file pone.0105412.s001.doc]

**Table S1** Quantification of chemical markers selected in the ^1^H NMR spectra of DHI samples in Phase I

| **No.** | **Batch** | **Mean (*n^a^* =3 ) ± SD (mg mL-1)** | | | | | | | | | | | | |
| --- | --- | --- | --- | --- | --- | --- | --- | --- | --- | --- | --- | --- | --- | --- |
|  |  | **Valine** | **Threonine** | **Alanine** | **Pyroglutamate** | **Succinate** | **Asparagine** | **Malonate** | **Fructose** | **Glucose** | **Rutinose** | **Salvianic acid** | **Procatechuic acid** | **Procatechuic aldehyde** |
| 1 | 12051069 | 0.1777 ± 0.0031 | 0.2402 ± 0.0031 | 0.1465 ± 0.0012 | 1.159 ± 0.002 | 0.3872 ± 0.0016 | 1.694 ± 0.020 | 1.0087 ± 0.0031 | 1.627 ± 0.016 | 4.312 ± 0.043 | 0.9671 ± 0.0026 | 1.7340 ± 0.0098 | 0.1800 ± 0.0035 | 0.13133 ± 0.00058 |
| 2 | 12051076 | 0.1607 ± 0.0045 | 0.1980 ± 0.0120 | 0.1314 ± 0.0032 | 0.994 ± 0.049 | 0.350 ± 0.012 | 0.476 ± 0.022 | 0.9047 ± 0.0023 | 1.387 ± 0.037 | 3.896 ± 0.062 | 0.859 ± 0.015 | 1.633 ± 0.060 | 0.1697 ± 0.0042 | 0.1217 ± 0.0042 |
| 3 | 12051006 | 0.1660 ± 0.0073 | 0.2226 ± 0.0073 | 0.1356 ± 0.0047 | 1.017 ± 0.017 | 0.3634 ± 0.0070 | 1.569 ± 0.065 | 0.9835 ± 0.0167 | 1.585 ± 0.013 | 4.138 ± 0.040 | 0.940 ± 0.034 | 1.718 ± 0.032 | 0.1803 ± 0.0064 | 0.1260 ± 0.0052 |
| 4 | 12051007 | 0.1642 ± 0.0024 | 0.2189 ± 0.0020 | 0.1371 ± 0.0012 | 0.9953 ± 0.0058 | 0.3571 ± 0.0021 | 1.533 ± 0.013 | 0.9768 ± 0.0071 | 1.545 ± 0.038 | 4.070 ± 0.083 | 0.9320 ± 0.0058 | 1.721 ± 0.045 | 0.2023 ± 0.0021 | 0.1400 ± 0.0052 |
| 5 | 12061020 | 0.1698 ± 0.0025 | 0.2353 ± 0.0039 | 0.1429 ± 0.0021 | 1.118 ± 0.014 | 0.371 ± 0.012 | 1.592 ± 0.027 | 0.990 ± 0.014 | 1.601 ± 0.038 | 4.326 ± 0.022 | 0.963 ± 0.017 | 1.740 ± 0.017 | 0.1867 ± 0.0075 | 0.1340 ± 0.0030 |
| 6 | 12061036 | 0.1772 ± 0.0074 | 0.2380 ± 0.0011 | 0.1451 ± 0.0028 | 1.130 ± 0.051 | 0.383 ± 0.017 | 1.582 ± 0.017 | 0.985 ± 0.027 | 1.524 ± 0.015 | 4.172 ± 0.094 | 0.986 ± 0.012 | 1.730 ± 0.020 | 0.1937 ± 0.0025 | 0.13133 ± 0.00058 |
| 7 | 12061042 | 0.1698 ± 0.0023 | 0.2178 ± 0.0029 | 0.1417 ± 0.0033 | 1.156 ± 0.051 | 0.420 ± 0.026 | 1.456 ± 0.043 | 1.042 ± 0.030 | 1.664 ± 0.053 | 4.302 ± 0.065 | 1.080 ± 0.016 | 1.767 ± 0.048 | 0.1800 ± 0.0053 | 0.1353 ± 0.0031 |
| 8 | 12061050 | 0.1625 ± 0.0046 | 0.2264 ± 0.0052 | 0.1372 ± 0.0035 | 1.249 ± 0.120 | 0.3148 ± 0.0017 | 1.434 ± 0.033 | 0.948 ± 0.016 | 1.565 ± 0.032 | 4.122 ± 0.062 | 0.910 ± 0.019 | 1.696 ± 0.032 | 0.1670 ± 0.0035 | 0.1190 ± 0.0017 |
| 9 | 12071001 | 0.1442 ± 0.0011 | 0.2061 ± 0.0037 | 0.1216 ± 0.0020 | 0.8966 ± 0.0089 | 0.2797 ± 0.0053 | 1.350 ± 0.010 | 0.8530 ± 0.0057 | 1.493 ± 0.011 | 3.909 ± 0.022 | 0.8083 ± 0.0087 | 1.684 ± 0.024 | 0.1600 ± 0.0035 | 0.1210 ± 0.0052 |
| 10 | 12071045 | 0.1520 ± 0.0028 | 0.2253 ± 0.0025 | 0.1369 ± 0.0026 | 0.9404 ± 0.0063 | 0.2949 ± 0.0015 | 1.586 ± 0.016 | 0.9216 ± 0.0047 | 1.5346 ± 0.0088 | 4.265 ± 0.030 | 0.9781 ± 0.0048 | 1.567 ± 0.028 | 0.1513 ± 0.0021 | 0.1153 ± 0.0032 |
| 11 | 12081050 | 0.1573 ± 0.0012 | 0.2227 ± 0.0031 | 0.1326 ± 0.0006 | 1.1160 ± 0.0087 | 0.3123 ± 0.0021 | 1.475 ± 0.015 | 0.9367 ± 0.0093 | 1.6122 ± 0.052 | 4.429 ± 0.077 | 0.888 ± 0.0078 | 1.622 ± 0.043 | 0.1587 ± 0.0040 | 0.1153 ± 0.0021 |
| 12 | 12091001 | 0.1537 ± 0.0038 | 0.2183 ± 0.0075 | 0.1297 ± 0.0025 | 0.9447 ± 0.015 | 0.2943 ± 0.0065 | 1.437 ± 0.045 | 0.902 ± 0.011 | 1.542 ± 0.059 | 4.271 ± 0.029 | 0.864 ± 0.022 | 1.564 ± 0.013 | 0.1520 ± 0.0082 | 0.1100 ± 0.0017 |
| 13 | 12091018 | 0.1477 ± 0.0049 | 0.2177 ± 0.0076 | 0.1243 ± 0.0038 | 0.945 ± 0.020 | 0.2977± 0.0051 | 1.491 ± 0.045 | 0.890 ± 0.014 | 1.5782 ± 0.0067 | 4.464 ± 0.020 | 0.832 ± 0.020 | 1.638 ± 0.021 | 0.1533 ± 0.0015 | 0.1130 ± 0.0026 |
| 14 | 12111024 | 0.1500 ± 0.0030 | 0.2223 ± 0.0021 | 0.1350 ± 0.0030 | 0.9280 ± 0.0060 | 0.2910 ± 0.0010 | 1.582 ± 0.029 | 0.9093 ± 0.0047 | 1.501 ± 0.047 | 4.562 ± 0.040 | 0.9653 ± 0.0051 | 1.7993 ± 0.0045 | 0.20833 ± 0.00058 | 0.15533 ± 0.00058 |
| 15 | 12111031 | 0.1600 ± 0.0026 | 0.2277 ± 0.0025 | 0.1400 ± 0.0020 | 0.7947 ± 0.0035 | 0.2710 ± 0.0017 | 1.430 ± 0.024 | 0.9210 ± 0.0030 | 1.469 ± 0.065 | 4.597 ± 0.048 | 0.9677 ± 0.0080 | 1.544 ± 0.017 | 0.1673 ± 0.0032 | 0.1260 ± 0.0010 |
| 16 | 12111040 | 0.1620 ± 0.0020 | 0.2270 ± 0.0036 | 0.1403 ± 0.0005 | 0.8040 ± 0.011 | 0.2790 ± 0.0040 | 1.452 ± 0.021 | 0.953 ± 0.036 | 1.557 ± 0.096 | 4.539 ± 0.297 | 0.985 ± 0.025 | 1.63 ± 0.11 | 0.176 ± 0.022 | 0.130 ± 0.012 |
| 17 | 12121039 | 0.1493 ± 0.0005 | 0.2010 ± 0.0017 | 0.1196 ± 0.0006 | 0.8957 ± 0.0029 | 0.2746 ± 0.0021 | 1.4570 ± 0.0070 | 0.879 ± 0.036 | 1.47 ± 0.11 | 4.39 ± 0.25 | 0.853 ± 0.029 | 1.685 ± 0.026 | 0.1713 ± 0.0015 | 0.1230 ± 0.0026 |
| 18 | 12121055 | 0.1530 ± 0.0036 | 0.2040 ± 0.0036 | 0.1230 ± 0.0020 | 0.8840 ± 0.0017 | 0.27267 ± 0.00058 | 1.502 ± 0.030 | 0.8680 ± 0.0017 | 1.460 ± 0.063 | 4.218 ± 0.074 | 0.8590 ± 0.0026 | 1.717 ± 0.024 | 0.1743 ± 0.0031 | 0.12867 ± 0.00058 |
| 19 | 13011001 | 0.1533± 0.0035 | 0.2097 ± 0.0038 | 0.1233 ± 0.0025 | 0.9027 ± 0.0081 | 0.2737 ± 0.0012 | 1.5143 ± 0.030 | 0.8700 ± 0.0052 | 1.445 ± 0.034 | 4.559 ± 0.034 | 0.8437 ± 0.0076 | 1.6790 ± 0.0087 | 0.16567 ± 0.00058 | 0.1247 ± 0.0021 |
| 20 | 13011006 | 0.1623± 0.0015 | 0.2040 ± 0.0017 | 0.1277 ± 0.0015 | 0.8873 ± 0.0067 | 0.3013 ± 0.0021 | 1.509 ± 0.018 | 0.8793 ± 0.0072 | 2.887 ± 0.062 | 4.285 ± 0.064 | 0.8853 ± 0.0071 | 1.6883 ± 0.0071 | 0.1720 ± 0.0044 | 0.1320 ± 0.0030 |
| 21 | 13011017 | 0.1663± 0.0035 | 0.2027 ± 0.0051 | 0.1330 ± 0.0017 | 0.897± 0.012 | 0.3280 ± 0.0030 | 1.537 ± 0.026 | 0.9063 ± 0.0097 | 1.451 ± 0.030 | 4.465 ± 0.053 | 0.943 ± 0.016 | 1.7517 ± 0.0084 | 0.1963 ± 0.0040 | 0.1490 ± 0.0053 |
| 22 | 13011023 | 0.1610± 0.0044 | 0.2053 ± 0.0055 | 0.1273 ± 0.0031 | 0.914± 0.012 | 0.2983 ± 0.0035 | 1.498 ± 0.044 | 0.897 ± 0.012 | 1.492 ± 0.051 | 4.281 ± 0.041 | 0.871 ± 0.014 | 1.672 ± 0.025 | 0.1740 ± 0.0010 | 0.1227 ± 0.0023 |
| 23 | 13011030 | 0.1503± 0.0023 | 0.1987± 0.0047 | 0.1210 ± 0.0017 | 0.860± 0.012 | 0.2487 ± 0.0074 | 1.421 ± 0.030 | 0.890 ± 0.042 | 1.478 ± 0.019 | 4.13 ± 0.13 | 0.896 ± 0.039 | 1.578 ± 0.074 | 0.1643 ± 0.0058 | 0.1173 ± 0.0051 |
| 24 | 13011043 | 0.1687± 0.0032 | 0.1967± 0.0051 | 0.1320± 0.0020 | 0.9700± 0.0052 | 0.3290 ± 0.0036 | 1.437 ± 0.031 | 0.9257 ± 0.0051 | 1.418 ± 0.053 | 4.210 ± 0.072 | 1.002 ± 0.010 | 1.600 ± 0.0070 | 0.1983 ± 0.0038 | 0.1453 ± 0.0015 |
| 25 | 12031007 | 0.1667± 0.0076 | 0.2397± 0.0031 | 0.1417± 0.0025 | 0.962± 0.010 | 0.4010 ± 0.0040 | 1.537 ± 0.034 | 0.9783 ± 0.0057 | 1.5142 ± 0.0057 | 3.874 ± 0.050 | 0.860 ± 0.022 | 1.652 ± 0.022 | 0.1707 ± 0.0032 | 0.1130 ± 0.0010 |

*^a^* The number of samples (*n*) used for quantification.

**Table S2** Quantification of 13 chemical markers selected in the ^1^H NMR spectra of DHI samples in Phase II

| **No.** | **Batch** | **Mean (*n^a^* =3 ) ± SD (mg mL-1)** | | | | | | | | | | | | |
| --- | --- | --- | --- | --- | --- | --- | --- | --- | --- | --- | --- | --- | --- | --- |
|  |  | **Valine** | **Threonine** | **Alanine** | **Pyroglutamate** | **Succinate** | **Asparagine** | **Malonate** | **Fructose** | **Glucose** | **Rutinose** | **Salvianic acid** | **Procatechuic acid** | **Procatechuic aldehyde** |
| 1 | 12081064 | 0.1633 ± 0.0015 | 0.2267 ± 0.0029 | 0.13833 ± 0.00058 | 1.3050 ± 0.0085 | 0.3140 ± 0.0026 | 1.3917 ± 0.0025 | 0.944 ± 0.013 | 1.530 ± 0.055 | 4.110 ± 0.016 | 0.905 ± 0.018 | 1.739 ± 0.031 | 0.1723 ± 0.0049 | 0.1347 ± 0.0032 |
| 2 | 12111048 | 0.2257 ± 0.0025 | 0.3203 ± 0.0032 | 0.1980 ± 0.0017 | 1.07033 ± 0.00058 | 0.3863 ± 0.0015 | 2.070 ± 0.014 | 0.977 ± 0.016 | 1.551 ± 0.059 | 4.133 ± 0.023 | 0.934 ± 0.033 | 1.715 ± 0.040 | 0.1983 ± 0.0067 | 0.1370 ± 0.0052 |
| 3 | 12111056 | 0.2257 ± 0.0042 | 0.3093 ± 0.0055 | 0.1960 ± 0.0017 | 1.1457 ± 0.0067 | 0.3870 ± 0.0017 | 2.000 ± 0.023 | 0.9643 ± 0.040 | 1.516 ± 0.055 | 4.066 ± 0.010 | 0.926 ± 0.011 | 1.730 ± 0.027 | 0.1860 ± 0.0087 | 0.1333 ± 0.0032 |
| 4 | 12121047 | 0.2180 ± 0.0046 | 0.302 ± 0.015 | 0.1753 ± 0.0035 | 1.306 ±  0.016 | 0.3983 ± 0.0055 | 2.163 ± 0.041 | 0.966 ± 0.020 | 1.494 ± 0.051 | 4.155 ± 0.054 | 0.9100 ± 0.0035 | 1.7400 ± 0.0052 | 0.1880 ± 0.0069 | 0.1300 ± 0.0017 |
| 5 | 13011037 | 0.2380 ± 0.0078 | 0.290 ± 0.010 | 0.1913 ± 0.0055 | 1.347 ±  0.041 | 0.440 ± 0.012 | 2.147 ± 0.070 | 0.946 ± 0.040 | 1.491 ± 0.072 | 4.210 ± 0.030 | 0.927 ± 0.022 | 1.728 ± 0.043 | 0.177 ± 0.015 | 0.1243 ± 0.0070 |
| 6 | 110402 | 0.1553 ± 0.0047 | 0.2217 ± 0.0078 | 0.1197 ± 0.0042 | 0.866 ±  0.020 | 0.2630 ± 0.0082 | 1.311 ± 0.058 | 0.839 ± 0.022 | 1.606 ± 0.058 | 3.987 ± 0.087 | 0.674 ± 0.012 | 1.550 ± 0.038 | 0.1633 ± 0.0040 | 0.1040 ± 0.0030 |
| 7 | 110404 | 0.1547 ± 0.0021 | 0.2213 ± 0.0040 | 0.1193 ± 0.0023 | 0.828 ±  0.021 | 0.1197 ± 0.0064 | 1.313 ± 0.059 | 0.872 ± 0.029 | 1.608 ± 0.050 | 4.025 ± 0.039 | 0.688 ± 0.024 | 1.548 ± 0.031 | 0.1620 ± 0.0013 | 0.1033 ± 0.0025 |
| 8 | 110405 | 0.1490 ± 0.0046 | 0.2163 ± 0.0051 | 0.1140 ± 0.0030 | 0.775 ±  0.016 | 0.2890 ± 0.0050 | 1.2297 ± 0.0047 | 0.82 ± 0.19 | 1.56 ± 0.10 | 3.90 ± 0.13 | 0.706 ± 0.023 | 1.467 ± 0.031 | 0.1590 ± 0.0053 | 0.0997 ± 0.0015 |
| 9 | 110408 | 0.1543 ± 0.0015 | 0.2263 ± 0.0025 | 0.1187 ± 0.0015 | 0.7747 ± 0.0093 | 0.2757 ± 0.0040 | 1.264 ± 0.035 | 0.853 ± 0.015 | 1.660 ± 0.071 | 4.051 ± 0.075 | 0.752 ± 0.030 | 1.536 ± 0.016 | 0.1700 ± 0.0026 | 0.1047 ± 0.0032 |
| 10 | 110410 | 0.1457 ± 0.0021 | 0.2143 ± 0.0015 | 0.1170 ± 0.0007 | 0.8000 ± 0.0061 | 0.3057 ± 0.0042 | 1.346 ± 0.018 | 0.8397 ± 0.0045 | 1.5707 ± 0.1150 | 3.8363 ± 0.1402 | 0.715 ± 0.014 | 1.6097 ± 0.0031 | 0.1683 ± 0.0031 | 0.1067 ± 0.0012 |
| 11 | 12081037 | 0.1700 ± 0.0028 | 0.2165 ± 0.0035 | 0.1430 ± 0.0028 | 1.148 ±  0.025 | 0.4040 ± 0.0085 | 1.474 ± 0.029 | 1.025 ± 0.025 | 1.58 ± 0.089 | 4.46 ± 0.11 | 1.078 ± 0.023 | 1.630 ± 0.040 | 0.1620± 0.0057 | 0.1160 ± 0.0057 |

*^a^* The number of samples (*n*) used for quantification.
